# Lifestyle Modifies the Diabetes-Related Metabolic Risk, Conditional on Individual Genetic Differences

**DOI:** 10.3389/fgene.2022.759309

**Published:** 2022-03-09

**Authors:** Jisu Shin, Xuan Zhou, Joanne T. M. Tan, Elina Hyppönen, Beben Benyamin, S. Hong Lee

**Affiliations:** ^1^ Australian Centre for Precision Health, University of South Australia Cancer Research Institute, University of South Australia, Adelaide, SA, Australia; ^2^ UniSA Allied Health and Human Performance, University of South Australia, Adelaide, SA, Australia; ^3^ National Cancer Center, Goyang-si, South Korea; ^4^ Vascular Research Centre, Heart and Vascular Health Program, Lifelong Health Theme, South Australian Health and Medical Research Institute, Adelaide, SA, Australia; ^5^ Adelaide Medical School, University of Adelaide, Adelaide, SA, Australia; ^6^ UniSA Clinical and Health Sciences, University of South Australia, Adelaide, SA, Australia; ^7^ South Australian Health and Medical Research Institute, Adelaide, SA, Australia

**Keywords:** GxE interaction, lifestyle, metabolic disease, precision medicine, type 2 diabetes

## Abstract

Metabolic syndrome is a group of heritable metabolic traits that are highly associated with type 2 diabetes (T2DM). Classical interventions to T2DM include individual self-management of environmental risk factors, such as improving diet quality, increasing physical activity, and reducing smoking and alcohol consumption, which decreases the risk of developing metabolic syndrome. However, it is poorly understood how the phenotypes of diabetes-related metabolic traits change with respect to lifestyle modifications at the individual level. In the analysis, we used 12 diabetes-related metabolic traits and eight lifestyle covariates from the UK Biobank comprising 288,837 white British participants genotyped for 1,133,273 genome-wide single nucleotide polymorphisms. We found 16 GxE interactions. Modulation of genetic effects by physical activity was seen for four traits (glucose, HbA1c, C-reactive protein, systolic blood pressure) and by alcohol and smoking for three (BMI, glucose, waist–hip ratio and BMI and diastolic and systolic blood pressure, respectively). We also found a number of significant phenotypic modulations by the lifestyle covariates, which were not attributed to the genetic effects in the model. Overall, modulation in the metabolic risk in response to the level of lifestyle covariates was clearly observed, and its direction and magnitude were varied depending on individual differences. We also showed that the metabolic risk inferred by our model was notably higher in T2DM prospective cases than controls. Our findings highlight the importance of individual genetic differences in the prevention and management of diabetes and suggest that the one-size-fits-all approach may not benefit all.

## Introduction

Diabetes mellitus is a metabolic disease normally caused by high blood glucose levels, which can lead to complications in kidneys, eyes, and the nervous system (Rolo and Palmeira, 2006). Currently, it is one of the top 10 leading causes of death in the world (Heron, 2018), highlighting the importance of improved strategies on prevention and management. Type 2 diabetes mellitus (T2DM), which accounts for more than 90% of all cases of diabetes (Chatterjee et al., 2017), is known to be more polygenic than other types of diabetes (Kramer et al., 2003; Jiang et al., 2019). T2DM is often comorbid with other complex diseases, such as cardiovascular diseases (Giovannucci et al., 2010; Martín-Timón et al., 2014), and metabolic syndrome is highly associated with increasing the risk for both T2DM and cardiovascular diseases (Haffner, 2006; Abou Ziki and Mani, 2016). Metabolic syndrome is a group of traits that causes metabolic diseases, such as diabetes. Diabetes-related metabolic traits can include glucose, hemoglobin A1c (HbA1c), C-reactive protein (CRP), body mass index (BMI), cholesterols, and blood pressure (BP) (Sabatti et al., 2009).

Metabolic abnormalities and diabetes risk are affected by genetic factors (Kong et al., 2015). A recent genome-wide association study (GWAS) identified 143 genetic variants associated with T2DM that shed light on the etiology of the disease (Xue et al., 2018). However, the identified genetic variants explain only a small proportion of phenotypic variance (Maher, 2008; Manolio et al., 2009; Xue et al., 2018), which is unlikely to accurately predict the individual genetic (or polygenic) risk of T2DM in early life stages (Janssens and van Duijn, 2008). A whole-genome approach using all common single nucleotide polymorphisms (SNPs), instead of using a few genome-wide significant SNPs, has been proposed as a new promising approach for polygenic risk prediction (Yang et al., 2010; Fernando et al., 2017; Khera et al., 2019). Recently, it has been shown that the accuracy of polygenic risk prediction can be increased further when using advanced statistical models and designs (Maier et al., 2018; Lloyd-Jones et al., 2019; Truong et al., 2020; Zhou et al., 2020).

In addition to the genetic factors, T2DM risks are also increased by environmental conditions, such as unhealthy diet and physical inactivity (Dendup et al., 2018). Therefore, T2DM preventions and interventions include improving diet quality, increasing physical activity, and reducing smoking and alcohol consumption. These interventions are often uniformly recommended for people with high metabolic risk irrespective of their response to these interventions. However, this one-size-fits-all approach may be inefficient because it does not consider individual genetic differences (Schork, 2015; Prasad and Groop, 2019; Philipson, 2020). In fact, it is little known how the T2DM risk in response to lifestyle modification can vary with respect to individuals’ genotypes, i.e., genotype-by-environment (GxE) interaction, and it is poorly understood how the information of GxE interaction can be incorporated in a T2DM intervention. It is unlikely that the changed genetic effects by lifestyle modification are in the same direction and magnitude for all people; therefore, the lifestyle modification should be tailored to each individual, considering individual genetic difference, i.e., personalized intervention.

In this study, we applied a whole-genome approach (Ni et al., 2019) to estimate the genetic and nongenetic effects on 12 diabetes-related metabolic traits modulated by eight lifestyle covariates. We show that the direction and magnitude of metabolic risk in response to the level of lifestyle covariates vary depending on individual genetic differences, i.e., phenotypic plasticity of the risk. We also show that the predicted metabolic risk of T2DM prospective cases is significantly higher than controls. We conclude that a paradigm shift in intervention approaches for T2DM is required to account for individual differences, which are realized as precision medicine afforded by the increased availability of genomic data (e.g., UK Biobank) and advanced computational models. This novel approach will enable more accurate treatments and preventions of T2DM.

## Methods

### Ethical Statement

The UK Biobank’s scientific protocol and operational procedures were reviewed and approved by the North West Multi-Centre Research Ethics Committee (MREC), National Information Governance Board for Health & Social Care (NIGB), and Community Health Index Advisory Group (CHIAG). The access to the UK Biobank data was approved by the UK Biobank based on the application 14575 [“Whole-genome approaches for dissecting (shared) genetic architecture and individual risk prediction of complex traits in human populations”]. Research ethics were approved by the University of South Australia Human Research Ethics Committee (HREC).

### Participants

The UK Biobank consists of more than 500,000 individuals, recruited from 22 assessment centers across the UK between March 2006 and October 2010 (Sudlow et al., 2015). The participants were recruited when they were between 37 and 73 years old (Ollier et al., 2005), and all information used in this study are derived from information collected during the baseline survey.

### Phenotypic Data

As outcomes, we used the information of diabetes-related metabolic traits, including glucose, HbA1c, total cholesterol (TC), LDL cholesterol (LDL), HDL cholesterol (HDL), CRP, sodium and potassium in urine, BMI, waist–hip ratio (WHR), and systolic and diastolic BP with details on assay and measures given in Supplementary Material (Supplementary Note S1). Lifestyle covariates were obtained by self-report and included age at recruitment, alcohol intake frequency (ALC), smoking status (SMK), and metabolic equivalent task (MET) minutes per week for walking, moderate, vigorous, total activities and healthy dietary scores (Supplementary Note S2).

### Genotypic Data

This study used the UK Biobank genotypic data, which comprise 92,693,895 SNPs genotyped from 488,377 participants. In preliminary quality controls, we excluded individuals and SNPs that did not meet the following criteria from the UK Biobank data. At the individual level, we excluded individuals who were not white British (to reduce the effect of population stratification), have a missing rate ≥5%, have a gender mismatch between self-reported and genetic data, and with putative sex chromosome aneuploidy. One individual from a pair, which has a genomic relationship larger than .05, was randomly selected and excluded. Furthermore, individuals who were population outliers (i.e., not within ±6 standard deviations from the first and second principal components) were excluded. At the SNP level, SNPs with an information score less than .6, with SNP missing rate less than 95%, with Hardy–Weinberg equilibrium *p*-value less than 0.0001, and with minor allele frequency less than 1% were excluded. Duplicated SNPs were also removed. The ratio of discordant SNPs between the initial and second released individuals in the UK Biobank data was calculated, and an additional 29 individuals who had a discordance rate more than 0.05 were excluded. We only used HapMap3 SNPs from the quality-controlled data, which are of high quality and well calibrated to dissect the genetic architecture of complex diseases (Ripke et al., 2013; Tropf et al., 2017). After quality controls, the cleaned data include 1,133,273 SNPs and 288,837 participants.

Due to the usage of the individual level of genotypic data, which demands high computational resources, we further divided samples into six groups. Of the total samples, 91,472 individuals from the first release of these selected individuals were divided into two groups. Meanwhile, 197,365 individuals from the second release were divided into four groups. Meta-analysis estimates and *p*-values across the six groups were reported.

### Data Analysis

#### Adjustment for the Phenotypes of Main Traits and Lifestyle Covariates.

For the interaction analyses, the phenotypes of the main traits (Supplementary Table S1) were adjusted for demographics, assessment center, genotype measurement batch, and population structure measured by the first 10 principal components (PCs) (Jin et al., 2011). Demographic variables included sex, year of birth, income, education, and Townsend deprivation index. The education variable was obtained following Okbay et al. (2016), and the details are in Supplementary Table S2. For each interaction analysis, the covariate in the interaction model was also used to adjust the phenotypes of the main trait, which was necessary to avoid any spurious interaction signals due to correlations between the main trait and the covariate (Robinson et al., 2017; Ni et al., 2019). In addition to these key variables for the adjustment, other lifestyle covariates not in the model could be considered, depending on their relevance to the main trait. We note that the glucose was further adjusted for fasting time, which is the interval between the last food or drink and blood sample being taken.

When a lifestyle covariate was used as the second trait in a bivariate model (see Supplementary Note S3), the phenotypes of the second trait were also adjusted for potential confounders including demographics, batch, center, and the first 10 PCs. In addition to these potential confounders, other lifestyle covariates were possibly considered, depending on their relevance to the second trait. The distribution of adjusted phenotypes of lifestyle covariates are shown in Supplementary Figure S1.

For the diabetes-related metabolic traits in the main analyses, an additional quality control (QC) was applied to the adjusted phenotypes to exclude outliers that are outside the three standard deviations in either direction from the mean of the phenotypic data (Osborne, 2010). The adjusted and QCed phenotypes were further transformed using a rank-based inverse normal function to satisfy the underlying assumption of the model, i.e., the normality assumption (see Supplementary Figure S2). Note that these steps are essential to prevent spurious interaction signals (Robinson et al., 2017; Ni et al., 2019). The number of individual records remained after these processes (adjustment, outlier QC, and transformation) are listed in Supplementary Table S3.

#### Statistical Models

The overall workflow for the designed experiment is in Supplementary Figure S3. We used a multivariate reaction norm model (MRNM) that can estimate both GxE and residual-by-environment (RxE), simultaneously (Ni et al., 2019), where RxE indicates the nongenetic effects that are modulated by the lifestyle covariates. Data analyses were performed using MTG2 software (Lee and van der Werf, 2016). In the main analyses, there were four models, i.e., a null model without any interactions, models with GxE or RxE interaction only, and a full model jointly fitting GxE and RxE interactions. The maximum likelihoods from the four models were compared to test if there was significant interaction (see Supplementary Figure S3). A significance *p*-value threshold was set at 5.21E-04 (= .05/96) after Bonferroni correction to account for 96 tests in total. We declared a significance if the *p*-value was lower than the significance threshold and the sum of estimated variances of GxE and RxE (i.e., $\sigma_{g_{1}}^{2}$ and/or $\sigma_{\tau_{1}}^{2}$ ) was nonnegative to avoid estimated interaction effects out of the legitimate parameter space. The model description for MRNM can be found in Supplementary Note S3.

#### Model Comparisons to Detect Interaction Effects

Based on the model comparison using four different MRNMs (Supplementary Note S3), five different interaction effects can be tested (Supplementary Figure S3; Supplementary Table S4). The restricted maximum likelihood values obtained from the four models were used to assess their model fits (Ni et al., 2019). The significance of interaction effects was determined based on the *p*-values from likelihood ratio chi-squared tests comparing the full and reduced models. The five kinds of interaction effects are 1) overall interaction detected from the comparison between the null and full models, which includes both GxE and RxE interaction, noting that the overlapping section represents the collinearity between estimated GxE and RxE interactions (Ni et al., 2019); 2) GxE interaction detected from the comparison between the null and GxE only models, which is not corrected for the collinearity with RxE interaction; 3) RxE interaction detected from the comparison between the null and RxE only models, which is not corrected for the collinearity with GxE interaction; 4) orthogonal GxE interaction detected from the comparison between the RxE only and full models, which is corrected for the collinearity with RxE interaction; and 5) orthogonal RxE interaction detected from the comparison between GxE and full models, which is corrected for the collinearity with GxE interaction (Supplementary Figure S3). It is noted that, while overall interactions are important, it is of interest to disentangle between GxE and RxE interactions that are without collinearity, referred to as orthogonal GxE or RxE interaction (Supplementary Figure S3).

#### Predicted Phenotypes (Risk) of Metabolic Traits

Based on the full model (see Supplementary Note S3), the expected phenotypes for each individual, comprising estimated additive genetic ( $\hat{\alpha_{0}}$ ), GxE interaction ( $\hat{\alpha_{1}}$ ) and RxE interaction effects ( $\hat{\tau_{1}}$ ), can be written as

$$\hat{y}=\hat{\alpha_{0}}+\hat{\alpha_{1}}\cdot c+\hat{\tau_{1}}\cdot c$$ (1)

where $\hat{y}$ is the predicted phenotypes and c is the standardized lifestyle covariate that with a mean of zero and a standard deviation of one. To be consistent across traits, we used the full model to predict the phenotypes for all traits.

Furthermore, we derived the trajectory of the predicted phenotypes across different levels of lifestyle covariates in each of the 96 analyses (12 traits x 8 covariates). For this, individuals were divided into three groups, that is, the top, middle, and bottom 20% groups according to the estimated interaction effects (the sum of GxE and RxE effects, i.e., $\;\hat{\alpha_{1}}+\hat{\tau_{1}}$ ). In addition, each of the three groups was further stratified into two groups, T2DM prospective cases and controls. Note that we restricted to use incident (i.e., prospective) cases only, according to ICD-10 information (code E11) to avoid any effects of T2DM status on metabolic risk (Supplementary Note S1). We estimated the intercept and slope of a linear model regressing the predicted phenotypes on the covariate for each group.

The values of intercepts and slopes were averaged over the 69 analyses that showed significant signals for both GxE and RxE interactions. The averaged values of intercepts and slopes might represent the overall relationship between metabolic risk and lifestyle covariates. In this process, we considered making favorable and unfavorable directions consistent across the main traits and covariates to facilitate a better interpretation in line with metabolic risks on T2DM. For example, the sign of HDL, physical activity, and healthy diet values were switched when analyzing phenotypes so that the direction of favorableness for these variables is consistent with other variables (glucose, HbA1c, TC, LDL, CRP, sodium, potassium, BMI, WHR, systolic BP, diastolic BP, Age, ALC, SMK). To test whether the difference between the groups with cases and controls is statistically significant, we did a paired *t*-test.

In addition to the comparison using 69 analyses with the significant overall interactions, we performed the same analyses using 16 analyses that showed significant signals for orthogonal GxE interaction and 58 analyses for orthogonal RxE interaction. The predicted phenotypes were grouped as same as the overall interaction according to the estimated of GxE or RxE interaction effects (i.e., $\hat{\alpha_{1}}$ or $\hat{\tau_{1}}$ ).

#### Heritability

We calculated the heritability using the estimated genetic and residual variances from the null model (i.e., multivariate GREML) or interaction model (MRNM), which can be expressed as

$$h^{2}=\;\frac{\sigma_{\alpha_{0}}^{2}}{\sigma_{\alpha_{0}}^{2}+\sigma_{\tau_{0}}^{2}}$$

where $\sigma_{\alpha_{0}}^{2}$ is the main genetic variance and $\sigma_{\tau_{0}}^{2}$ is the residual variance. This estimation assumes the environmental homogeneity of the study sample.

#### Causality Analysis

As complementary analyses, we used CAUSE software (Morrison et al., 2020), which is a newly proposed Mendelian randomization method, to detect the causal effects of lifestyle covariates on T2DM metabolic traits. In causality analyses, the GWAS summary statistics of pruned SNPs were used to infer the causality and its significance, following the instruction of CAUSE. The same genotype and phenotype data as in the main interaction analyses were used in the causality analyses. We note that the phenotypes of the metabolic traits used as outcomes in causality analyses were required not to be adjusted for the covariate used as the exposure (i.e., lifestyle covariate) because CAUSE tests the association of the first moment (mean), different from MRNM that estimates the second moment (variance) across covariate values.

## Results

### Interactions

We tested if the genetic (GxE) and nongenetic effects (RxE) of 12 diabetes-related metabolic traits are modulated by eight lifestyle covariates (see *Methods*) and found that 69 out of 96 tests showed significant signals for the interactions when comparing the full and null models (Supplementary Figure S4). The significant *p*-values were obtained from the likelihood ratio tests after Bonferroni correction. As shown in Supplementary Figure S4, the genetic and nongenetic effects of glucose and HbA1c, which are highly associated with T2DM (Supplementary Table S5), are shown to be significantly modulated by all eight lifestyle factors. CRP, which is a well-known biomarker of inflammation, is also significantly altered by all lifestyle covariates.

**FIGURE 1:**
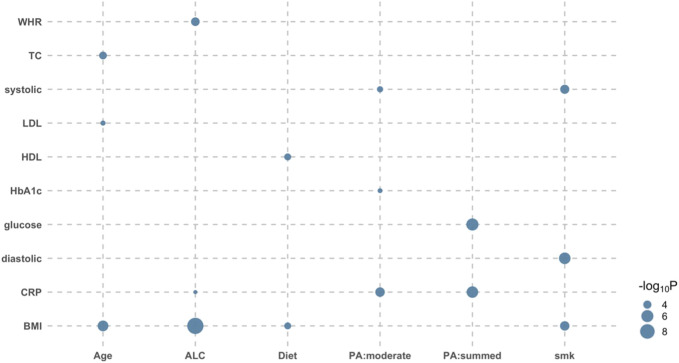
Bubble plot of *p*-values indicating significant orthogonal GxE interactions. There were 16 significant orthogonal GxE interactions when testing the genetic effects of 12 diabetes-related metabolic traits modulated by eight covariates. Likelihood ratio tests were used to compare the full model with the RxE only model for each of six independent data sets, and *p*-values were meta-analyzed using the Fisher method. The size of dot reflects its significance; the bigger the more significant. For example, the BMI-ALC pair is most significant (*p*-value = 1.86E-10), indicating that ALC significantly modulates genetic effects of BMI phenotypes. WHR, waist-hip ratio; TC, Total cholesterol; systolic, systolic blood pressure; sodium, sodium in urine; potassium, potassium in urine; LDL, low density lipoprotein cholesterol; HDL, high density lipoprotein cholesterol; HbA1c, haemoglobinA1C; CRP, C reactive protein; BMI, body mass index; Age, Age at recruitment; ALC, alcohol intake frequency; smk, smoking status; PA:summed, a summed physical activity; PA:walk, physical activity walking; PA:moderate, physical activity moderate intensity; PA:vigorous, physical activity vigorous intensity; Diet, healthy dietary scores.

It is of interest to disentangle the modulated genetic effects from nongenetic effects, i.e., GxE effects orthogonal to RxE effects. When comparing the full model with the RxE only model, there were 16 tests showing significant orthogonal GxE interactions after the Bonferroni correction (see *Methods*) (Figure 1; Supplementary Table S6). Specifically, the genetic effects of glucose were shown to be modulated by the level of physical activity (*p*-value = 7.18E-07). Strong modulations of genetic effects by physical activity were observed for HbA1c and CRP (*p*-values < 4.66E-04). Although there was no evidence of orthogonal interaction between physical activity and BMI, other lifestyle factors significantly modulated the genetic effects of BMI (e.g., *p*-value = 1.86E-10 for ALC-BMI) (Figure 1).

We also compared the full model with GxE only model to assess orthogonal RxE interaction effects and found 58 significant signals out of 96 tests (Supplementary Figure S5). The significance of RxE interaction was generally stronger than that of GxE interaction. For glucose and HbA1c, which are closely related to T2DM, most of the lifestyle factors had significant modulation effects, captured by orthogonal RxE interaction. It is remarkable that there were significant interaction signals for CRP that were consistently observed across all lifestyle factors with *p*-values ranging from 4.32E-96 to 5.51E-12. In the analyses of BMI, a strong risk factor of T2DM, it was shown that the non-genetic effects (Zhou and Lee, 2021) of BMI were significantly modulated by ALC and physical activity.

A number of orthogonal RxE interactions found in this study can be supported by a causality analysis, using CAUSE software (Morrison et al., 2020) (Supplementary Figure S6). For example, CAUSE analysis showed significant causal effects of lifestyle factors on phenotypes for pairs of ALC-HbA1c, ALC-TC, ALC-HDL and diet-sodium, which also appeared to be significant for the orthogonal RxE interaction (Supplementary Figure S5). In addition, the significant causal relationship of each pair of ALC-BMI, ALC-WHR, and SMK-WHR can be partly explained by the orthogonal GxE interaction (Figure 1) or combined GxE and RxE interactions (Supplementary Figure S2).

MRNM allows individually different responses to a lifestyle modification, which cannot be modeled in standard additive models. To demonstrate this property of MRNM, we plotted predicted phenotypes (see Eq. 1 in *Methods*) against the standardized values of lifestyle factors for three groups (the top, middle, and bottom 20%) stratified according to the magnitude of estimated GxE interaction (Supplementary Figure S7). This shows that the expected phenotypes in response to the modification of lifestyle factors can be different among individuals, and the slope of phenotypes is positive, zero, or negative for the top, middle, or bottom group, respectively (Supplementary Figure S7). This demonstrates that individual genetic difference should be carefully considered in a lifestyle modification, i.e., an intervention of metabolic risk.

**FIGURE 2:**
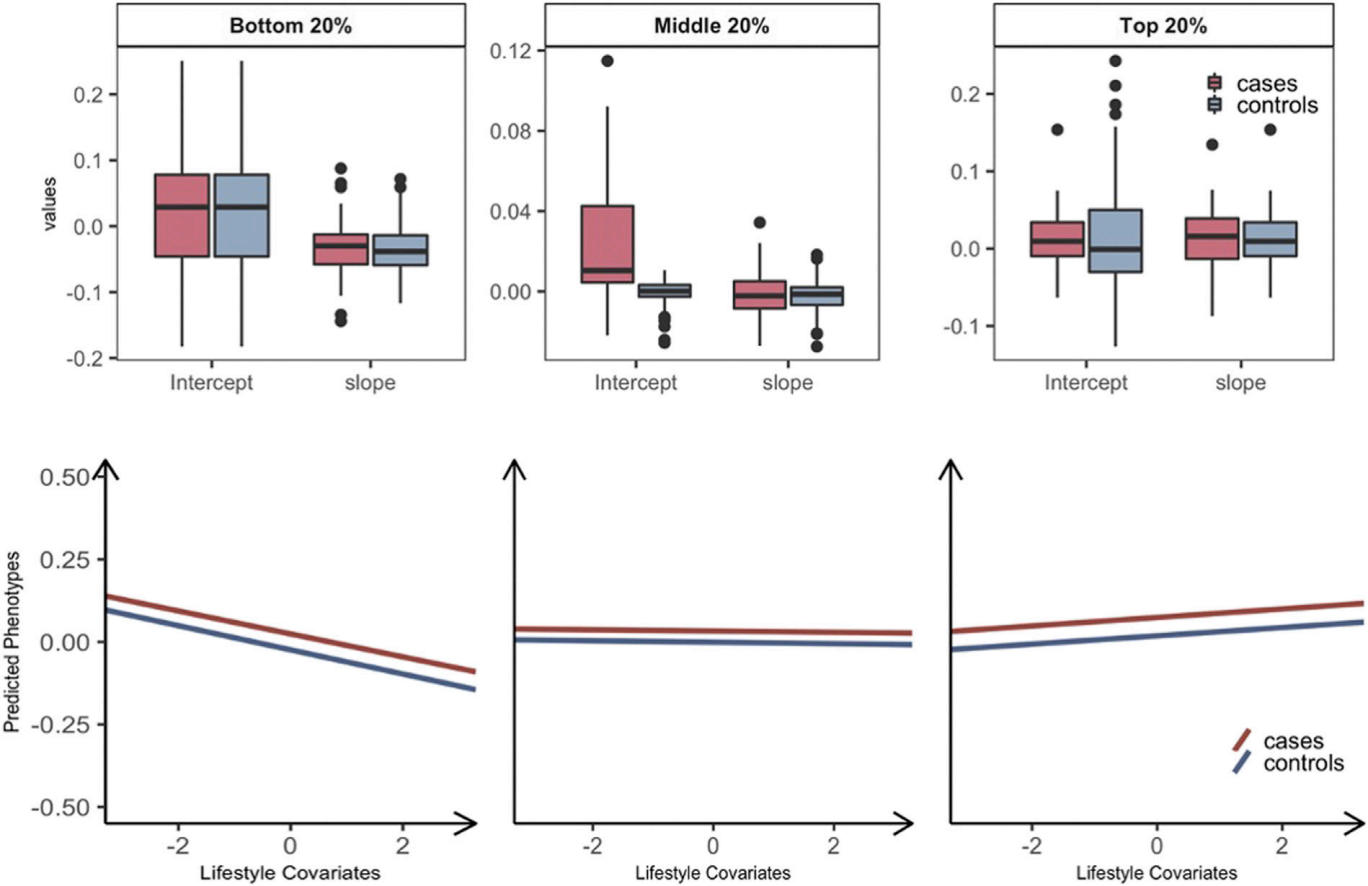
Plasticity of diabetes-related metabolic traits of 69 significant overall interactions in response to the level of lifestyle effects by grouping T2DM prospective cases and controls. Individuals are stratified into three groups: the bottom, middle, and top 20% groups according to estimated GxE and RxE interactions from the full model (i.e., $\sigma_{\alpha_{1}}^{2}+\sigma_{\tau_{1}}^{2}$ ). We further classified individuals into T2DM prospective cases (red) and controls (blue) in each of three groups, hence, six groups in total. To compare the differences in trajectories between with T2DM cases and controls, a linear model regressing the phenotype of main trait on standardized lifestyle measures was used to estimate the intercept and slope for each of six groups. The values of intercepts and slopes were averaged over the 69 analyses that showed significant signals for both GxE and RxE interactions. The averaged values of intercepts and slopes represent the overall relationship between metabolic risk and lifestyle covariates, where we considered making favorable and unfavorable directions consistent across the main trait and covariates to facilitate a better interpretation in line with metabolic risks on T2DM (see *Methods*). Boxplots for each group are represented to show the differences in terms of the calculated intercepts and slopes, and the mean of values for intercepts and slopes were represented as regression lines. The mean and standard error of intercepts and slopes across the analyses of the 69 pairs with significant overall interactions were estimated to assess if there is any significant difference of the mean between cases and control in each of the three groups: the bottom, middle, and top 20% groups. There were no significant differences between cases and controls in the mean of slopes in all three groups. The mean of intercepts is significantly different between cases and controls in all three groups (*p*-values = 2.25E-07, 6.98E-10, and 1.68E-09). The arrows on both axes in linear regression figures indicate an unfavorable direction in regard to T2DM health.

We further stratified each of the three groups into T2DM prospective cases and controls, hence, six groups in total. The intercept and slope of regressing the predicted phenotypes on the standardized lifestyle measures were estimated for each of the six groups (e.g., Supplementary Figure S8). We applied this approach to the 69 pairs with significant overall interactions (Figure 2) and calculated the mean and standard error of intercepts and slopes across all pairs, to assess if there was any significant difference between prospective cases and controls in each of the bottom, middle, and top 20% groups (Figure 2). It was shown that the intercepts of prospective cases were significantly higher than those of controls in all three groups (paired *t*-test *p*-value = 2.25E-07, 6.98E-10, and 1.68E-09), indicating that prospective cases were likely to have higher metabolic risk than controls. However, the slope of predicted risks was not significantly different between prospective cases and controls, showing that the change of metabolic risk in response to lifestyle modification is invariant across prospective cases and controls. Similar results were observed when considering GxE or RxE interaction only in that the intercepts were higher for prospective cases than controls (Supplementary Figures S9 ,S10). We note that, when using the model of RxE interaction only, the slope was significantly steeper for prospective cases than controls in each of the three groups, suggesting that the metabolic risk of prospective cases is more sensitive to lifestyle modification, compared with controls (Supplementary Figure S10), which was, however, not observed when using the model of GxE interaction only.

### Heritability

**FIGURE 3:**
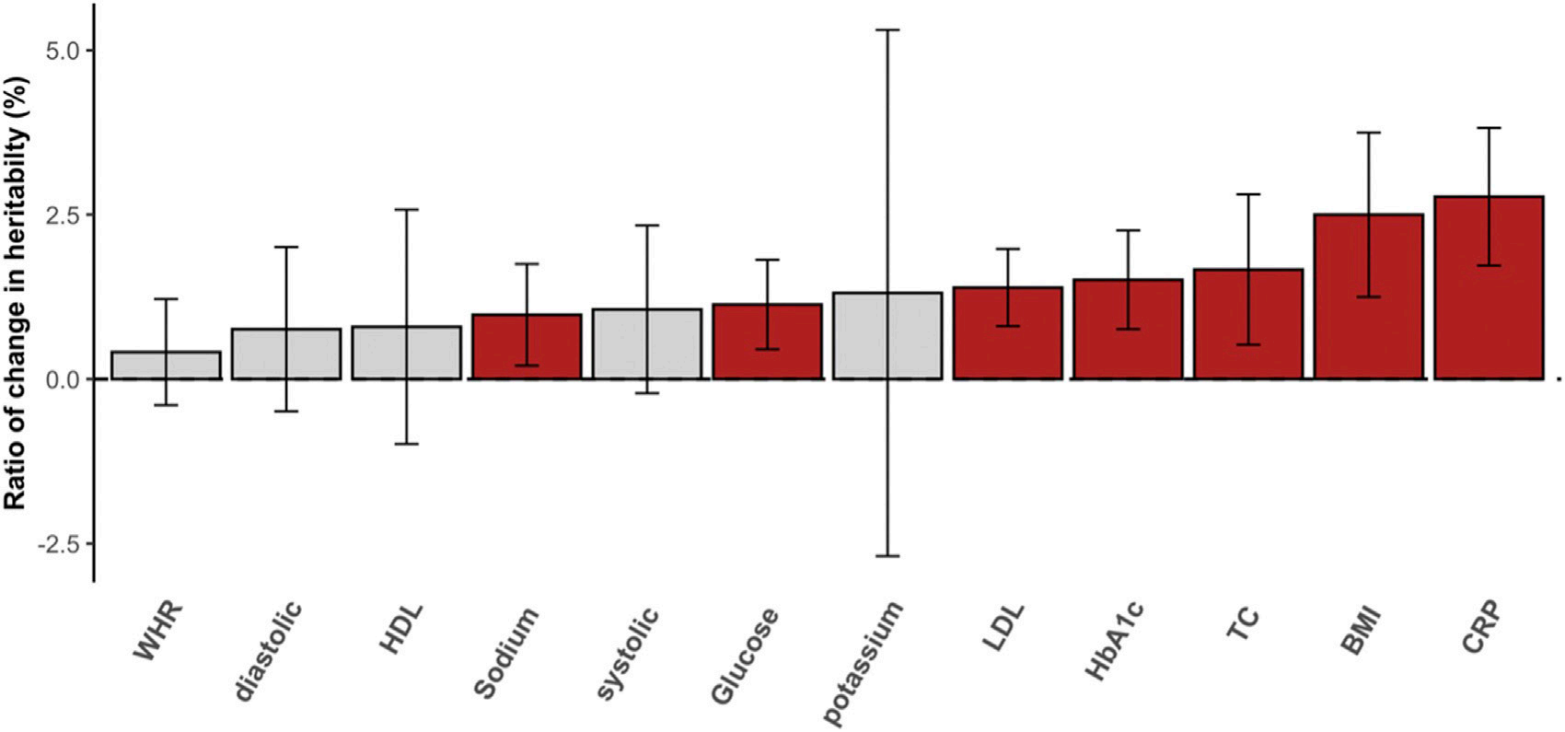
The ratio of change in SNP heritability. The differences between heritability estimates from the GREML and MRNM were represented as the ratio of change in heritability (%). Each bar indicates the differences in heritability, and the ratio was calculated as ( $h_{MRNM}^{2}-h_{GREML}^{2}$ )/ $h_{GREML}^{2}$ , where $h_{MRNM}^{2}$ and $h_{GREML}^{2}$ indicate the estimated heritability from MRNM and GREML, respectively. Therefore, the positive ratio denotes that estimate of MRNM is higher than that of GREML. The vertical line is the 95% confidence interval of the ratio averaged over the analyses of each trait with significant overall interaction. Traits with significant difference between MRNM and GREML are colored red. The interaction analyses of 69 pairs with significant overall interactions were used in the heritability comparisons. WHR, waist-hip ratio; diastolic, diastolic blood pressure; HDL, high density lipoprotein cholesterol; sodium, sodium in urine; LDL, low density lipoprotein cholesterol; potassium, potassium in urine; TC, total cholesterol; HbA1c, haemoglobinA1C; BMI, body mass index; CRP, C reactive protein; systolic, systolic blood pressure.

Heritability estimation can be biased if nonnegligible interaction effects are not properly modeled. In the standard additive model (e.g., GREML), the variance attributed to unmodeled interactions can be partitioned as residual variance, which results in underestimated heritability, i.e., so-called still-missing heritability (Wray and Maier, 2014). This is evident for diabetes-related metabolic traits (Figure 3) for which the ratio of change in heritability for each trait was positive and not negligible. For example, the estimated heritability of CRP increased by 2.5% when significant interactions were considered appropriately. In the comparison of glucose and HbA1c, which are most relevant to T2DM, we observed more than 1.5% of heritability changes, and this supports the hypothesis that a fraction of still-missing heritability in metabolic traits is due to interaction effects being unaccounted for. The estimated variances for the main genetic and residual effects from GREML and MRNM were compared, showing that the residual variance was mostly overestimated (hence, underestimated heritability) in the GREML (Supplementary Figure S11).

## Discussion

It is well established that phenotypic changes in diabetes-related metabolic traits, such as glucose and HbA1c, are associated with lifestyle modifications (Harding et al., 2001; Nakanishi et al., 2003; Rafalson et al., 2009), which has motivated lifestyle interventions for the prevention and treatment of T2DM (Eriksson and Lindgrde, 1991; Knowler et al., 2002; Shin et al., 2012). However, it remains unknown if these interventions should be applied uniformly to everyone (i.e., a one-size-fits-all approach) or tailored to individuals (i.e., precision health approach). In this study, we show that lifestyle modification can significantly alter the genetic and nongenetic effects of metabolic traits (i.e., GxE and RxE interactions), where the direction and magnitude of the alteration depend on individual differences in genetic information. This finding demonstrates that a more personalized approach is needed for T2DM intervention.

Previous studies have already indicated concerns about the inefficiency of the one-size-fits-all approach (Gill and Cooper, 2008; Pozzilli et al., 2014; Johansen, 2015; Schork, 2015). These concerns can be overcome by accounting for individual genetic difference. For example, it is desirable to know how the diabetes-related metabolic risk in response to lifestyle modifications varies across individuals according to their genotypes, and this knowledge will allow a personalized lifestyle intervention to T2DM that can be tailored to each individual need.

Our finding for significant genome-wide GxE interactions across diabetes-related metabolic traits is novel and can be applied in such personalized lifestyle interventions. To our knowledge, no whole-genome GxE interactions have been reported for diabetes-related metabolic traits. Previously, we reported significant GxE and RxE interactions for some metabolic traits, including BMI, BP, cholesterols, and WHR; however, GxE interaction could not be disentangled from RxE because of a small sample size (Ni et al., 2019; Zhou et al., 2020). In this study, we disentangled GxE interaction from RxE interaction using a large sample size for diabetes-related metabolic traits such as glucose, HbA1c and CRP that were not studied before. We also demonstrated the validity of the estimated RxE interactions, using CAUSE analyses (Morrison et al., 2020). RxE interaction can be also explained by environment-by-environment interactions (Zhou and Lee, 2021).

Unlike standard additive models, MRNM allows us to stratify samples into three groups (the top, middle, and bottom 20%) according to estimated individual GxE or/and RxE interaction effects. The patterns of the expected phenotypes of the metabolic traits were clearly distinct between the three groups. This shows that the one-size-fits-all approach may not be the best strategy in a T2DM intervention. Importantly, the predicted metabolic risk was significantly higher in T2DM prospective cases than controls. Interestingly, the phenotypic plasticity of metabolic risk in response to lifestyle modification is significantly different between prospective cases and controls only when considering nongenetic effects of metabolic risk (i.e., using the model with RxE interaction only).

We found that 69 significant signals out of 96 tests were detected for overall interactions, indicating that GxE and RxE interactions play a significant role in the etiology of T2DM. It is remarkable that 34.8% of the significant tests (24 out of 69) are strongly significant (*p*-value < 10E-100). However, these signals are mostly attributed to RxE interactions (Figure 1 vs. Supplementary Figure S5), which is also summarized in Supplementary Figure S3. To disentangle GxE from RxE, we adjusted the significance of GxE effects accounting for the collinearity with RxE and found 16 significant signals for orthogonal GxE interactions. Similarly, we found 58 significant signals for orthogonal RxE interactions. The smaller number of significant GxE interactions, compared with RxE interactions, is probably due to the fact that the power is smaller because a large number of genetic variants (>1 M) were involved in the interaction term (Sham and Purcell, 2014).

Although a causality analysis (CAUSE) was used to replicate some of our findings, we note that causality models (such as CAUSE or Mendelian randomization model) are different from MRNM in that they test associations among genetic instruments, exposure, and outcome at the phenotypic level using least squares or similar methods. MRNM, instead, adjusts and removes the phenotypic association because its main interest is to estimate the heterogeneity of genetic and nongenetic variance across different lifestyle values. Therefore, MRNM is probably robust to the assumptions to be made in the causality models (e.g., horizontal pleiotropic effects that are removed from the adjustment in MRNM). Nonetheless, consistent results from these two very different models can increase the reliability of the findings.

In summary, the modulation of diabetes-related metabolic risk in response to the level of lifestyle covariates was clearly observed, and its direction and magnitude varied depending on individual genetic differences. Interestingly, such genetic phenotypic plasticity was invariant across T2DM prospective cases and controls although the overall metabolic genetic risk is significantly higher in T2DM prospective cases than controls. Our findings highlight the importance of individual genetic differences in the prevention and management of diabetes and suggest that the one-size-fits-all approach may not benefit all.

## Data Availability

The genotype and phenotype data of the UK Biobank used in this study are publicly accessible through procedures described on its webpage (http://www.ukbiobank.ac.uk/using-the-resource). The source code for MTG version 2.15 is publicly available in https://sites.google.come/site/honglee0707/mtg2. The original contributions presented in the study are included in the article/Supplementary Material, further inquiries can be directed to the corresponding authors.
